# Photocatalytic dye degradation and antibacterial activity of gold nanoparticles: a DFT and machine learning study

**DOI:** 10.1039/d5ra08168h

**Published:** 2025-12-17

**Authors:** M. Yasmin Begum, Mukta Sharma, M. V. Arularasu, P. Vinitha, V. Vetrivelan, Geetha Kandasamy, A. Manikandan, Abinet Gosaye Ayanie, Lalitha Gnanasekaran, Ankush Mehta, Rupesh Gupta

**Affiliations:** a Department of Pharmaceutics, College of Pharmacy, King Khalid University Abha Saudi Arabia ybajen@kku.edu.sa; b Graduate Institute, Prospective Technology of Electrical Engineering and Computer Science, National Chin-Yi University Taichung 411030 Taiwan Republic of China muktashrm@ncut.edu.tw; c Sustainable Energy and Environment Research Unit, Department of Physiology, Saveetha Medical College, Saveetha Institute of Medical and Technical Science Chennai 602105 Tamil Nadu India arulrs597@gmail.com; d Department of Community Medicine, Saveetha Medical College, Saveetha Institute of Medical and Technical Science Chennai 602105 Tamil Nadu India vinithaprs18@gmail.com; e Department of Physics, Government College of Engineering Srirangam Tiruchirapalli 620012 Tamil Nadu India vetri.tpgit@gmail.com; f Department of Clinical Pharmacy, College of Pharmacy, King Khalid University Abha Saudi Arabia glakshmi@kku.edu.sa; g Department of Chemistry, Karpagam Academy of Higher Education Coimbatore 641021 Tamil Nadu India; h Centre for Material Chemistry, Karpagam Academy of Higher Education Coimbatore 641021 Tamil Nadu India manikandan.frsc@gmail.com; i Department of Mechanical Engineering, Adama Science and Technology University Adama 2552 Ethiopia abinet.gosaye@astu.edu.et; j Instituto de Alta Investigación, Universidad de Tarapacá Arica 1000000 Chile lalitha1887@gmail.com; k Marwadi University Research Center, Department of Mechanical Engineering, Faculty of Engineering & Technology, Marwadi University Rajkot 360003 Gujarat India ankush.mehta38@gmail.com; l Chitkara University Institute of Engineering and Technology, Chitkara University Rajpura 140401 Punjab India rupesh.gupta@chitkara.edu.in

## Abstract

This study explores the kinetic and thermodynamic factors influencing the photocatalytic degradation of methyl orange (MO) using gold nanoparticles (AuNPs) synthesized *via* a green route with *Acacia nilotica* extract as a reducing agent. The biosynthesized AuNPs exhibited excellent photocatalytic efficiency, achieving 92.5% degradation of MO within 10 minutes under visible light in the presence of NaBH_4_, and retained 88.4% activity after four successive cycles, demonstrating high reusability. Antibacterial activity was also confirmed against *Salmonella typhi* and *Lactobacillus acidophilus*. To validate and interpret the experimental outcomes, density functional theory (DFT) simulations were performed, examining the Fermi level, HOMO–LUMO gap, work function, topological properties (ELF, LOL), and thermal stability of AuNPs. In parallel, machine learning (ML) models, including XGBoost, LightGBM, and Neural Networks, were employed to predict electronic band gaps. The XGBoost model showed the highest accuracy with a root mean square error of 0.9878 and a mean squared error of 0.00035, while other models also produced results consistent with DFT values. This combined experimental, theoretical, and data-driven approach highlights the promise of AuNPs for efficient dye degradation and antibacterial applications, offering sustainable solutions for environmental remediation.

## Abstract 2

This study explores the kinetic and thermodynamic factors influencing the photocatalytic degradation of methyl orange (MO) using gold nanoparticles (AuNPs) synthesized *via* a green route with *Acacia nilotica* extract as a reducing agent.

## Graphical abstract

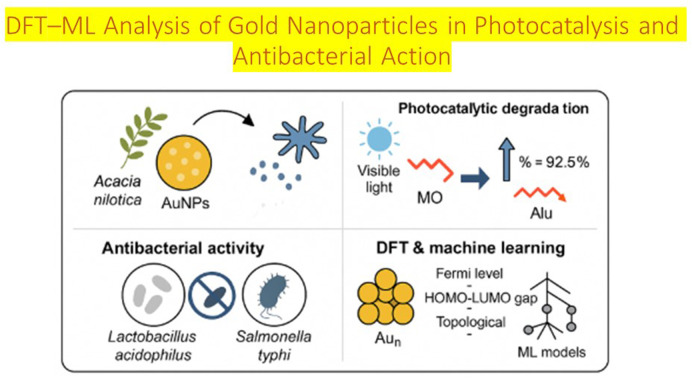

## 1. Introduction

In recent years, nanotechnology has been a fast-growing area of engineering and technology with distinct beneficial roles in the fields of biomedical research, food industry, health care, water remediation, cosmetics, and the development of optoelectronic materials.^1^ Metal nanoparticles have great attention owing to their applications in different technology fields, including catalysis, the chemical industry, gene delivery, biomedical sciences, sensing technologies, drug delivery, *etc.*^2–6^ NPs can be synthesized using physical, chemical, or electrochemical approaches; each has benefits and disadvantages. All these techniques (i) physical method using UV-induced irradiation, micro-emulsion and (ii) chemical methods comprise two steps: reduction (oxalic acid, sodium citrate, and citric acid) and stabilization (polymers, surfactant, PVA, dendrimers) of synthesis materials.^7–11^ The above methods can be expensive and time-consuming, and there are limitations on usage; chemical-reducing agents are often regarded as hazardous due to using many toxic substances to create negative environmental impact, *etc.*

Furthermore, controlling the shape and size of nanoparticles during the physicochemical method encounters common challenges by scientists. Moreover, green nanotechnology involves eco-friendly, convenient, and inexpensive NPs with a higher yield and stability than the physicochemical approach.^12^ Many green nanotechnology sources that normally exist in synthesizing metallic NPs using microorganisms, algae, plants, fungi, and yeast have been reported.^7,13–15^ Different biological agents require maintenance and cautious handling in the growing and culturing of these organisms. Furthermore, to some extent, it adds to the expenses. So, plant materials used to producing metal NPs.^16–18^

The discharge of effluents from industries containing dyes is creating environmental pollution due to their high threatens to humans and aquatic creatures. Therefore, the elimination of environmental pollution has become one of the widely researched topics. Methyl orange (MO) is a kind of water-soluble aromatic azo dye, a major class of synthetic organic compounds that are widely used in various consumer industries such as printing, leather, goods, pharmaceuticals, cosmetics, and so on. Water contamination by MO affects the water quality and causes mutagenic, genotoxic and carcinogenic effects on individuals and affects aquatic ecosystems.^19–21^ Consequently, the treatment of industrial wastewater is a necessity for a clean and green environment. Currently, several conventional techniques based on chemical, physical, biological, *etc.*, strategies have been employed for dye treatment. Among the variety of techniques, photocatalytic degradation under UV-Visible irradiation has attracted much interest in photodegradation of the MO into harmless products under mild reaction conditions. Also, photocatalysis is economically feasible and simple.^22^

As evidenced by the recent publications on AuNPs,^23,24^ they have special properties such as low toxicity, size-dependent properties, and catalysis; therefore, AuNPs are attractive today in various applications. AuNPs can effectively attack all types of viruses and bacteria compared to Pt and Ag NPs; further, compared with chemically prepared AuNPs, they are less hazardous and less toxic to the environment.^25^ Furthermore, AuNPs often have gaps or oxygen holes in the crystal structure due to electrons and oxygen missing; based on their unique properties, AuNPs play a substantial role in the nanotechnology field and have promising applications in drug delivery in bio-nanomedicine, biosensors, diagnostics, catalysis, bio-imaging.^26–28^ Hence, the focus has shifted to accomplish the bio-inspired green synthesis of AuNPs over *Acacia nilotica* leaf extract. *Acacia nilotica* is a tree native to Asia: it is commonly found in the dry land of various countries. This plant contains various phyto-constituents, including ellagic acid, kaempferol-7, tannins, flavonoids, and leucocyanidin.^29–31^ Owing to a bioactive functional group which is believed to have reduced the Au ion and then stabilized the AuNPs, furthermore this green synthesis approach provides AuNPs with better morphology. Based on the literature survey, AuNPs have been explored only for biomedical applications and degradation of MB, RhB, and Congo red, as per our knowledge. Apart from biomedical applications, photocatalytic dye degradation of MO and sensing of pollutants are equally important for developing dual-functional materials. AuNP synthesized from *Acacia nilotica* extract has never been investigated in earlier literature. Density functional theory using the LANL2DZ basis set and the B3LYP functional yields optimized structures of Au_*n*_NPs (*n* = 3, 4, 6, 8, 10, and 20).^32–35^ The band gap from the HOMO and the LUMO was utilized to determine the electronic characteristics and reactivity.^36,37^ Reactive sites on the clusters were identified and the charge distribution was visualized *via* MEP mapping. Lastly, a presentation and discussion of the observed data follow.

Thus, the objectives of this present study were: (i) to examine the green synthesis of AuNPs application towards the degradation of MO under 10 min visible light irradiation. Durability was verified by 4 successive cycles of re-use, which are also provided. The kinetics and possible mechanism of enhanced photocatalytic activity of AuNPs were also studied. (ii) The characteristics of AuNPs were analyzed by density functional theory and compared with machine learning experimental outcomes.

## 2. Experimental section

### 2.1. Materials

Tetrachloroauric Acid (HAuCl_4_·3H_2_O) and methyl orange were procured from SRL Chemical Pvt Ltd. Double-distilled water was used throughout the experiments. The chemicals were used as such without further purification.

### 2.2. Preparation of leaf extract

Leaves of *Acacia nilotica* were harvested from the local vicinity and surrounding villages in Thiruvallur, Tamil Nadu, and then dried for three weeks under direct sunlight. The well-dried leaves were finely pulverized to produce a powder. 20 g of *Acacia nilotica* leaf powder was boiled with 150 mL of distilled water for 30 min at a temperature of 70 °C. The extract is then filtered. The filtered extract functioned as a reducing agent in the synthesis of AuNPs.

### 2.3. Green synthesis of AuNPs

Approximately 60 mL of a 1 mM aqueous HAuCl_4_ solution was taken in a conical flask and kept under stirring at 800 rpm for 15 min at room temperature. 15 mL of *Acacia nilotica* leaf extract was poured and swirled continuously for 2 h in a dark environment. The colour of the Au precursor solution transitions from light yellow to reddish brown, indicating the growth of gold nanoparticles. The resultant solution was then dried using a rotary evaporator, and the resulting powdered AuNPs were used for future investigations. The phytoconstituents of *Acacia nilotica* leaves act as reducing agents that convert Au(iii) to Au (0) in the synthesis of AuNPs.

### 2.4. Characterization

A variety of characterization techniques, including High-resolution transmission Electron Microscopy (HR-TEM), UV-visible and Fourier-transform Infrared Spectroscopy (FT-IR), X-ray Diffraction (XRD) and Brunauer–Emmett–Teller (BET) Analysis, were instrumental in elucidating the physical attributes and potential utility of the synthesized AuNPs. UV-visible Spectroscopy (Model- UV-2450 spectrophotometer) was employed for verifying the formation of AuNPs and elucidating their size, concentration, and optical absorption properties, which are crucial for their photocatalytic application. FT-IR analysis shed light on the surface functional groups that modulate the efficacy of photocatalytic degradation. The IR spectrum of AuNCs was measured by using a Bruker IR spectrophotometer in ATR mode. HR-TEM offered detailed views of the nanoparticles' size, shape, and morphology, which are vital for understanding their surface area and reactivity. These characterization techniques provide a detailed picture of the AuNPs physical characteristics and their impact on functional efficacy in photocatalytic applications.

### 2.5. Photocatalytic test

Gold nanoparticles catalytic activity was tested by determining the degradation efficiency of MO dye under visible light irradiation (300 W intensity with wavelength 420 nm). The dye and catalyst were placed into a quartz photocatalytic reactor, and air was blown continuously into the solution to ensure that the dye and catalyst were well mixed. The as-fabricated photocatalyst (2–10 mL) was mixed with 30 mL of an aqueous MO solution and 5 mL of NaBH_4_ (0.01M). Before irradiation, the above mixture was kept stirring for 30 min under dark conditions to achieve equilibrium between sorption of the dyes as well as the photocatalyst. Dye samples were constantly taken from the vessel and centrifuged. The degradation of the dye solution was tested with UV-visible spectrophotometer in the range of 200–800 nm every 2 min during a total irradiation time of 10 min. Additionally, to study the recycling of catalyst experiments, the same catalyst was charged for 4 continuous cycles; each time, the catalyst was filtered and washed (distilled water) to remove residual dye impurities and dried at 60 ^°^C after each cycle.

### 2.6. Computational (calculation) details

AuNPs Au_*n*_ (*n* = 3, 4, 6, 8, 10, and 20) were designed and analyzed for this work using the computer programs Gaussian 09 and Gaussview 6.0. The shapes of molecules were optimized using the LANL2DZ basis set and the B3LYP functional to identify the most stable versions. The B3LYP functional combined with the LANL2DZ ECP was selected because it has been extensively validated and widely employed in previous studies of Au clusters, showing reliable performance for geometries, bond lengths, and electronic properties. Benchmark reports also indicate that B3LYP/ECP combinations provide results comparable to higher-level methods for small and medium-sized Au clusters.^38–40^

The chemical properties of Au_*n*_NPs were determined using eqn (1)–(7), which include the electrophilicity index (*ω*), electronegativity (*χ*), hardness (*η*), softness (*S*), chemical potential (*µ*), and the ability of gas phases to accept electrons (*ω*^+^) and donate electrons (ω^−^).

1 *µ* = (*E*_HOMO_ + *E*_LUMO_)/2

2 *η*= (*E*_LUMO_ − *E*_HOMO_)/2

3 *χ* = −*µ*

4 *S* = 1/*η*

5 *ω* = (*E*_HOMO_ + *E*_LUMO_)^2^/4 × (*E*_LUMO_ − *E*_HOMO_)

6 *ω*^−^= (3 × *E*_HOMO_ − *E*_LUMO_)^2^/16 × η

7 *ω*^+^= (*E*_HOMO_ + 3 × *E*_LUMO_)^2^/16 × η

The localized orbital locator (LOL) and electron localized function (ELF) are determined by the multiwfn software.^41,42^

Additionally, the following eqn (1) was used to predict Gibbs free energy.^43^

8 Δ*G*^0^(298)= ∑(*ε*_0_+*G*_corr_)_Final_− ∑(*ε*_0_+*G*_corr_)_initial_

Here, the (*ε*_0_+*G*_corr_) express the overall value of the thermal and electronic free energy.

### 2.7. Machine learning model performance

Three advanced machine learning models were trained and evaluated: XGBoost, LightGBM, and MLP Regressor (Neural Network).

## 3. Results and discussions

### 3.1. Characterization studies of green synthesized AuNPs

The employment of *Acacia nilotica* for the synthesis and characterization of AuNPs leverages its rich phytochemical profile, making it an exemplary agent for green synthesis over other methods. The presence of bioactive compounds such as tannins, flavonoids, saponins, and phenolic compounds in *Acacia nilotica* act as reducing and stabilizing agents. This biochemical diversity facilitates the efficient conversion of gold ions into AuNPs while ensuring their stability and preventing aggregation. The antioxidant characteristics of compounds like flavonoids and phenolics enable them to act as potent reducing agents, promoting an environmentally friendly synthesis process devoid of harmful chemicals. Additionally, these phytochemicals provide a natural capping to the AuNPs, assisting in controlling their size and shape, essential for producing well-defined nanoparticles.

The sustainable and eco-conscious methodology of using *Acacia nilotica* adheres to the principles of green chemistry, utilizing renewable resources and minimizing environmental repercussions. The biocompatibility of the synthesized AuNPs paves the way for various biomedical applications, such as drug delivery and wound healing, attributed to their low toxicity levels. Furthermore, the versatility of these nanoparticles extends to their use in catalysis, sensing, and environmental remediation, highlighting the superior performance afforded by the phytochemicals in *Acacia nilotica*. The selection of this synthesis agent is vindicated not only by its potent phytochemical makeup and the biocompatibility and stability of the resultant nanoparticles but also by its widespread availability, establishing *Acacia nilotica* as a prime candidate for the green synthesis of AuNPs.

**Fig. 1:**
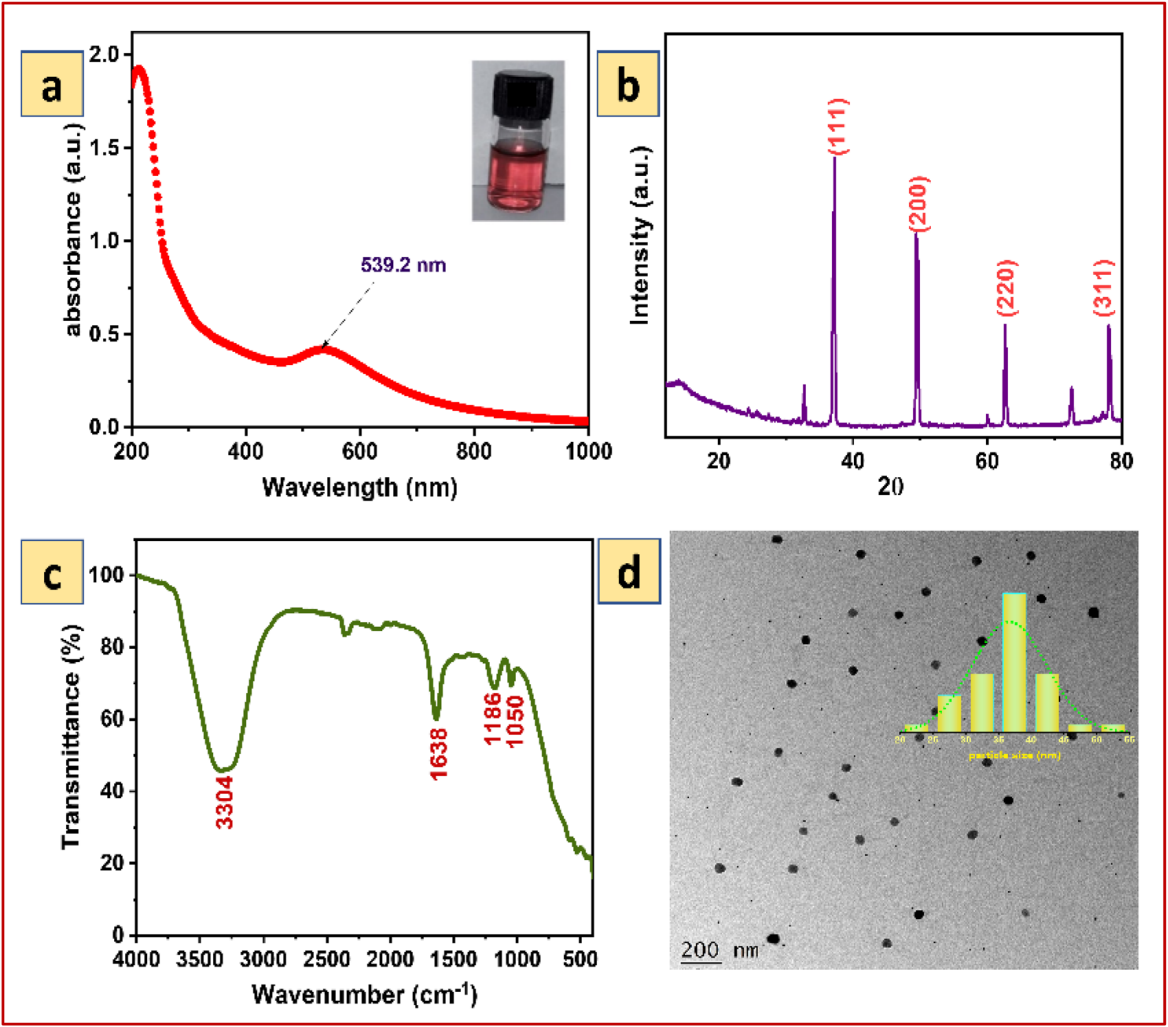
(a) UV-vis absorption spectrum (b) XRD pattern (c) FTIR spectrum and (d) TEM image with the size distribution histogram of AuNPs synthesized using *Acacia nilotica* as a reducing agent.

The aqueous leaf extract of *Acacia nilotica* added to the HAuCl_4_ solution led to a change of the mixture color from light yellow to purple-red, which confirms the reduction of Au^3+^ to AuNPs. The observed color changes occurred in the mixture due to the presence of the active molecules in the *Acacia nilotica* aqueous extract reducing the Au^3+^ ion to the stable AuNPs. Generally, the plasmonic peak of AuNPs appeared at 540 nm. The SRP peak at around 539.2 nm (shown in Fig. 1a) was similar to the previously reported AuNPs synthesized from the plant extract of *Curculigo orchioides*.^40^ Further, the presence of a single SRP peak provides strong evidence of the formation of size, morphology and collective oscillations of free electrons in the AuNPs, which was later confirmed using XRD, and TEM analysis.

XRD can be used to detect the phase identification and crystallinity of samples. The XRD spectrum of AuNPs is shown in Fig. 1b. The XRD peaks in 2*θ* of 38.1°, 44.3°, 64.5°, and 77.7° could be ascribed to the (111), (200), (220), and (311) crystallographic planes, thereby showing the cubic crystalline nature of the AuNPs, which was inconsistent with the face-centered cubic structure reference in JCPDS no. 04-0784. The existence of sharp peaks at the appropriate position as depicted in the literature reports of AuNPs ascribes the formation of AuNPs.

Unique stretching and bending vibrational bands in FT-IR can be considered a fingerprint to corroborate the functional groups present in a sample. The FTIR spectrum of AuNPs synthesized with the support of *Acacia nilotica* extract is illustrated in Fig. 1c. The peak seen at 3304 cm^−1^ corresponds to stretching of O–H bonding.^15^ The peak occurred at 1638 cm^−1^ attributed to the C=C stretching vibration from alkene molecules.^18^ The typical C–O stretching frequency of the carboxyl group was observed at 1186 and 1050 cm^−1^.^16^ The results suggest that the presence of phytochemical compounds such as flavonoids, terpenes, saponins, tannins, *etc.* present in *Acacia nilotica* act as stabilizing agents in the synthesis of AuNPs.^30^

The morphology of AuNPs can be identified through the transmission electron microscopy technique. Fig. 1d shows the well-dispersed particles with almost uniform sizes for synthesized AuNPs. The particles were generated in the size ranges of 20–60 nm with the average size range of 37.5 nm. The presence of capping agents such as flavonoids, terpenes, saponins, tannins, *etc.* present in *Acacia nilotica* over nanoparticles prevents them from further aggregation and promotes the formation of well-dispersed nanoparticles.

### 3.2. Photocatalytic degradation of methyl orange

**Fig. 2:**
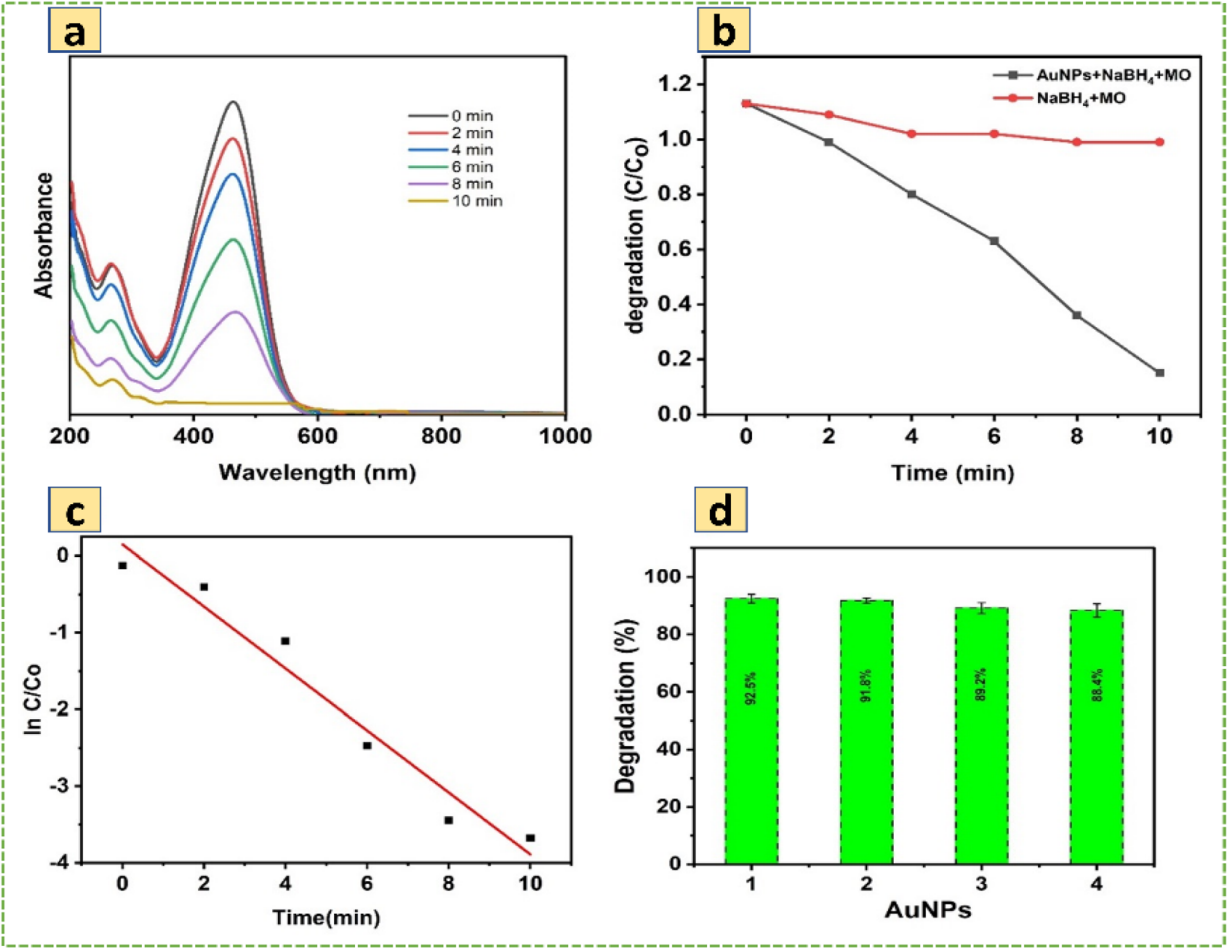
(a) Absorbance spectra of photo degradation for MO dye in the presence of AuNPs with NaBH_4_, (b) photodegradation activity of MO organic pollutant, (c) kinetics of MO by AuNPs, (d) recyclability of AuNP in MO dye removal.

The present study examines the photodegradation of MO dye in water, using green synthesized AuNPs in the presence of sodium borohydride as a catalyst, which was investigated by visible light irradiation. During the optimization process, the photocatalyst for effective dye degradation of MO was carried out by different amounts of AuNPs loading from 2 to 10 mL; we found 6 mL of photocatalyst provided the maximum dye degradation of MO, so 6 mL was chosen for the entire photocatalytic experiment. In the presence of AuNPs and NaBH_4_, the dye solution showed a gradual color change from orange to colorless, representing the completion of the dye degradation process. In this degradation process AuNPs acts as a photocatalyst and NaBH_4_ support to provide the necessary hydrogen to break MO dye. As depicted in Fig. 2a, two characteristic intense and broad adsorption peaks appeared at 267 nm and 584 nm, respectively, which are demonstrated in the UV-visible spectra during the evolution of photocatalytic degradation of MO. The absorption peak of 267 nm is due to π–π* transition of the aromatic moiety, and 584 nm is associated with the azo linkage (−N=N−) of MO.^43,44^ The entire MO dye photodegradation ended after 10 minutes while using AuNPs and NaBH_4_. It can be seen the unique absorption band at 584 nm gradually decreased with the degradation process. During the blank experiment, only the presence of NaBH_4_ and the reduction of MO dye were attempted in the absence of AuNPs. Catalysis was significantly slow because NaBH_4_ could not carry out the reduction efficiently. The photocatalytic degradation efficiency (Eqn (2)) and rate constants (Eqn (3)) of the AuNPs were calculated using the following equation,9
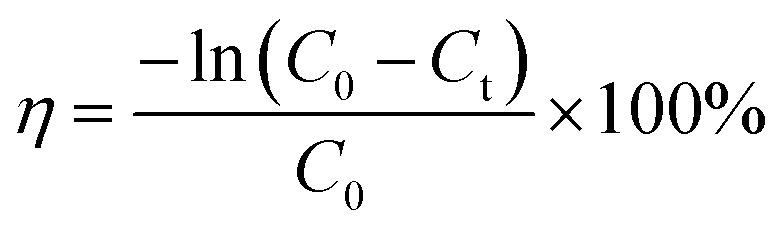
10
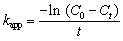
where, *C*_0_ and *C*_*t*_ represent the concentration of MO dye solution at the initial stage and at any time ‘*t*’ min. The dependencies of the photodegradation activity of MO under visible light irradiation are presented in Fig. 2b. According to Fig. 2b, AuNPs show a strong degradation process of MO under visible light irradiation for 10 min, and the degradation % was calculated to be 92.5%. Meanwhile, Fig. 2c indicates the plot of ln (*C*_*t*_/*C*_0_) against the time of substrates. After the induction time, a good linear correlation was obtained in the plot, indicating that MO degradation was followed by pseudo-first-order kinetics. The apparent kinetic reaction rate constant from the degradation of MO was 31.3 × 10^−3^ min^−1^.

The reusability of a photocatalyst is an important factor for its practical applications. Therefore, to explore the photocatalytic stability of AuNPs studies up to four continuous cycles, and results are indicated in Fig. 2d. As can be seen from the experimental results, the efficiency of the AuNPs slightly variation during the 1st (92.5%), 2nd (91.8%), 3rd (89.2%) and 4th (88.4%) cycles. The slight decrease in the catalytic activity probably occurred due to the loss of the catalyst during recollection. The photocatalyst could have good reusability and high activity after four successive cycles during the photocatalytic degradation of MO.

### 3.3. Antibacterial activity

**Fig. 3:**
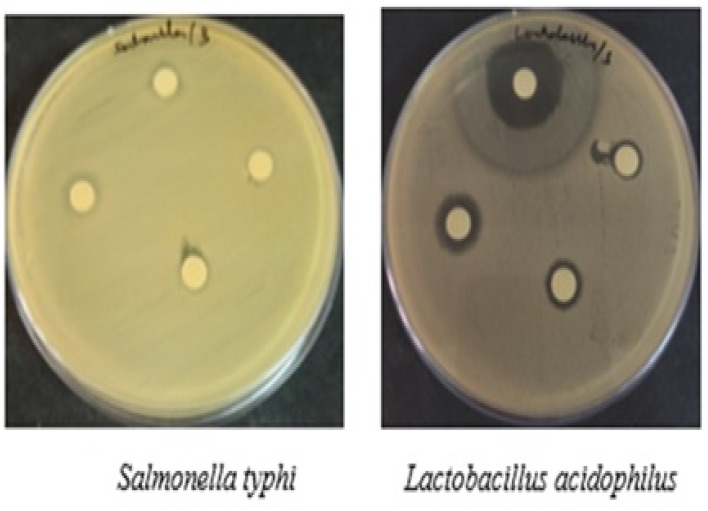
Antibacterial activity of AuNPs.

The antibacterial activity studies of AuNPs were examined through different concentration of the Gram-negative and Gram-positive organism as follows *Salmonella typhi* and *Lactobacillus acidophilus* bacteria. The results of inhibition zone diameter (nm) were showed in Fig. 3. The antibacterial effect of the AuNPs against *Salmonella typhi* bacterial strain MIC values found 11 mm, 10 mm and 9 mm respectively with at 100 µg mL^−1^, 75 µg mL^−1^, 50 µg mL^−1^ concentration. For the *Lactobacillus acidophilus* bacterial strain MIC values found 13 mm, 10 mm and 8 mm respectively with at 100 µg mL^−1^, 75 µg mL^−1^, 50 µg mL^−1^ concentration of AuNPs. The difference in the MIC value of Gram-negative and Gram-positive bacteria may be due to the physiochemical properties of the samples which is efficient penetrates through the cell wall and entrap the bacteria's biological process.

### 3.4. DFT studies

#### 3.4.1. Stable structure of Au_*n*_NPs analysis (*n* = 3, 4, 6, 8, 10 and 20)

**Fig. 4:**
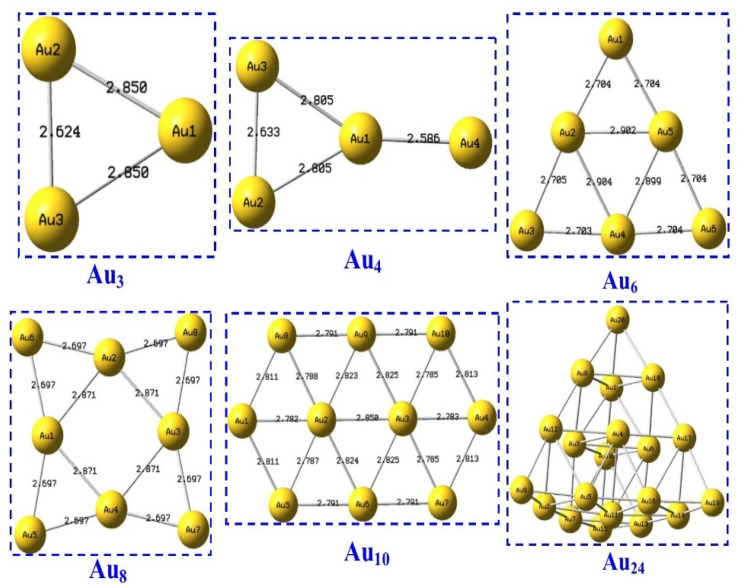
Optimized structure of Au_*n*_NPs (*n* = 3, 4, 6, 8 ,10, and 20).

The optimized structure of Au_*n*_NPs (*n* = 3, 4, 6, 8, 10 and 20) as shown in Fig. 4. A shorter bond suggests more electron attraction within the chemical system.

#### 3.4.2. Electronic and chemical reactivity parameters of Au_*n*_NPs (*n* = 3, 4, 6, 8, 10 and 20)

**Fig. 5:**
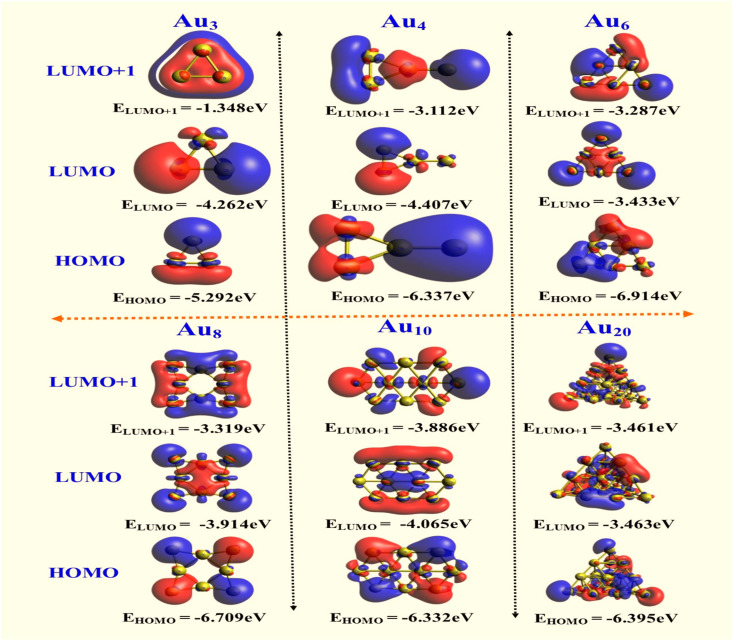
HOMO, LUMO and LUMO+1 plots of Au_*n*_NPs (*n* = 3, 4, 6, 8, 10, and 20) in gas phase.

Understanding the stability and reactivity of molecules requires an understanding of their orbitals. They are known as the HOMO and LUMO.^47,48^ The HOMO is the greatest energy level that can hold electrons, and the LUMO is the lowest energy level that can accept them. A closer look at these orbitals for the Au_*n*_ (*n* = 3, 4, 6, 8, 10, and 20) nanoparticles reveals possible chemical interactions. The energy difference between the HOMO and LUMO is known as the HOMO–LUMO gap, as seen in Fig. 5. By observing the features, significant details regarding the chemical activity of the Au_*n*_ nanoparticles can be determined. The intramolecular charge transfer that takes place within the molecules is explained by the lowest energy gap. Table 1 displays the HOMO–LUMO along with additional characteristics. For the Au_3_ cluster, the calculated HOMO–LUMO gap is 1.0 eV. With the addition of more Au atoms, the band gap varies according to the geometric arrangement of the Au_*n*_ clusters. Although the Au_6_ cluster shows a relatively higher band gap compared to the other studied clusters, all the optimized structures are electronically stable, as confirmed by their finite energy gaps. The electrophilicity index, which is calculated as a value of 22.2 eV in the gas phase and indicates how likely an atom or molecule is to take electrons from the environment, was higher in Au_3_NPs than in other clusters.

**Table 1:** Chemical descriptors of Au_*n*_NPs(*n* = 3, 4, 6, 8, 10 and 20) in gaseous phase computed at B3LYP/LANL2DZ

Parameters	Clusters					
	Au_3_	Au_4_	Au_6_	Au_8_	Au_10_	Au_20_
HOMO (eV)	−5.3	−6.3	−6.9	−6.7	−6.3	−6.4
LUMO (eV)	−4.3	−4.4	−3.4	−3.9	−4.1	−3.5
Ionization potential (IP)	5.3	6.3	6.9	6.7	6.3	6.4
Electron affinity (EA)	4.3	4.4	3.4	3.9	4.1	3.5
Energy gap *E*_g_ (eV)	1.0	1.9	3.5	2.8	2.3	2.9
Fermi level *E*_F_ (eV)	−4.8	−5.4	−5.2	−5.3	−5.2	−4.9
Work function *ϕ* (eV)	4.8	5.4	5.2	5.3	5.2	4.9
Dipole moment (debye)	1.412	3.632	0.003	0.003	0.001	0.001
Electronegativity *χ*	4.8	5.4	5.2	5.3	5.2	4.9
Chemical potential *µ*	−4.8	−5.4	−5.2	−5.3	−5.2	−4.9
Chemical hardness *η*	0.5	1.0	1.7	1.4	1.1	1.5
Chemical softness *S*	1.0	0.5	0.3	0.4	0.4	0.3
Electrophilicity index *ω*	22.2	15.0	7.7	10.2	11.9	8.3
Electronic charge	9.3	5.6	3.0	3.8	4.6	3.4
Electron donating capability (*ω*^−^.)	24.6	17.8	10.5	13.1	14.7	10.9
Electron accepting capability (*ω*^+^)	19.8	12.4	5.3	7.8	9.5	6.0

In addition to HOMO and LUMO, Fermi level (EF) is another important metric that should be discussed in this section. The Fermi level was identified using the following eqn (11).^45,46^11
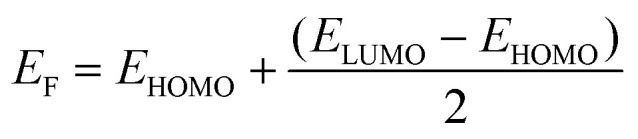


Fermi level of Au_*n*_NPs (*n* = 3, 4, 6, 8, 10, and 20) was determined in the gas phase at −4.8, −5.4, −5.2, −5.3, −5.2, and −4.9 eV, respectively, using the aforementioned approach. Any material that possesses the work function can be determined using *ϕ* = −*E*_F_. It calculates the absolute least amount of energy needed to detach an electron from any substance's surface. Any modification to the work function's size affects the device's overall performance and charge injection characteristics in electronic applications. Table 1 shows that the calculated *ϕ* values for Au_*n*_ (*n* = 3, 4, 6, 8, 10, and 20) nanoparticles in the gas phases are 4.8, 5.4, 5.2, 5.3, 5.2, and 4.9 eV, respectively. The prominent decrease in the work function (*ϕ*) is caused by the rearrangement of charges. This can result in the creation of an interfacial dipole, where the orientation and amplitude of the dipole moment can significantly reduce the work function, reducing the electron emission energy barrier.

The tendency of an electron to exit a molecule is measured by the chemical potential, or *µ*. The difficulty of losing one electron is indicated by a bigger negative value of *µ*. The Au_*n*_ NPs (*n* = 3, 4, 6, 8, 10, and 20) may have variable chemical potentials of −4.8, −5.4, −5.2, −5.3, −5.2, and −4.9 eV, respectively, due to the distribution of Au nanoparticles in various shapes and interactions with the environment. The electron distribution of a molecule is measured by its electronegativity, or *χ*. The higher electronegativity shown in the gas phase shows how effective they are as electron acceptors for Au_*n*_NPs (*n* = 3, 4, 6, 8, 10, and 20).

In the DFT framework, a molecule or chemical site's electrophilicity is measured by its electrophilicity index (*ω*). Molecules or sites with a higher *ω* are considered more electrophilic since they are more likely to receive electrons. Additionally, *ω*− and *ω*+ represent a molecule's ability to donate electrons and withdraw electrons, respectively. The current calculation indicates that the molecules under study have a significantly higher value of *ω*−than *ω*+.

Such small clusters are, however, widely used in theoretical studies because they effectively capture the local surface electronic structure, which predominantly governs catalytic and antibacterial activity. Catalysis on metal nanoparticles is generally controlled by surface atoms, low-coordination sites, and ligand–metal interactions, all of which are well-represented in small Au clusters.

Our approach is based on the established principle that changes in electronic properties (Fermi level, work function, HOMO–LUMO gap, charge distribution) induced by biomolecular capping occur locally at the surface and therefore can be reliably modelled using smaller clusters. These clusters allow us to systematically probe ligand-induced electronic modulation, which cannot be easily computed for 37.5 nm particles due to computational limitations.

The trends from the Au_*n*_ clusters—such as bandgap narrowing, enhanced charge transfer, and Fermi-level shifts—are directly correlated with the experimental observations of PL quenching, improved ROS generation, and enhanced catalytic degradation. Thus, although absolute values differ between nanoscale sizes, the qualitative electronic trends derived from small clusters provide meaningful mechanistic insights into the behavior of larger AuNPs.

In this way, the DFT results serve as a representative electronic model, enabling us to mechanistically connect the local electronic structure of the Au surface to the observed photocatalytic and antibacterial performance of the experimental nanoparticles.

The variation in the HOMO–LUMO gap from our DFT calculations directly supports the experimentally observed photocatalytic and antibacterial performance. A reduced HOMO–LUMO gap reflects enhanced electronic polarizability, improved charge-transfer capability, and lower excitation energy. These features facilitate more efficient generation of photo-induced charge carriers, which is consistent with the higher photocatalytic degradation efficiency of methyl orange observed experimentally.

Likewise, the lower gap indicates greater chemical softness and increased propensity for electron exchange with biological interfaces, which promotes reactive oxygen species formation and membrane disruption. This theoretical trend correlates well with the improved antibacterial activity recorded in our assays. Thus, the DFT-derived HOMO–LUMO variations substantiate and reinforce the experimental findings in both photocatalysis and antibacterial studies.

Electrical conductance (*σ*) is a crucial electronic parameter for sensitivity determination because of its exponential relationship with the energy gap (*E*_g_) is represented in eqn (12).^44^12
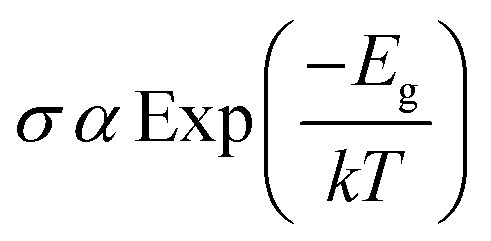
In this, Boltzmann constant and temperature were denoted by *K* and *T*, respectively. The link between electrical conductance (*σ*) and energy gap (*E*_g_) shows that the presence of a chemical compound will result in to an exponential rise in *σ* as *E*_g_ lowers.

**Fig. 6:**
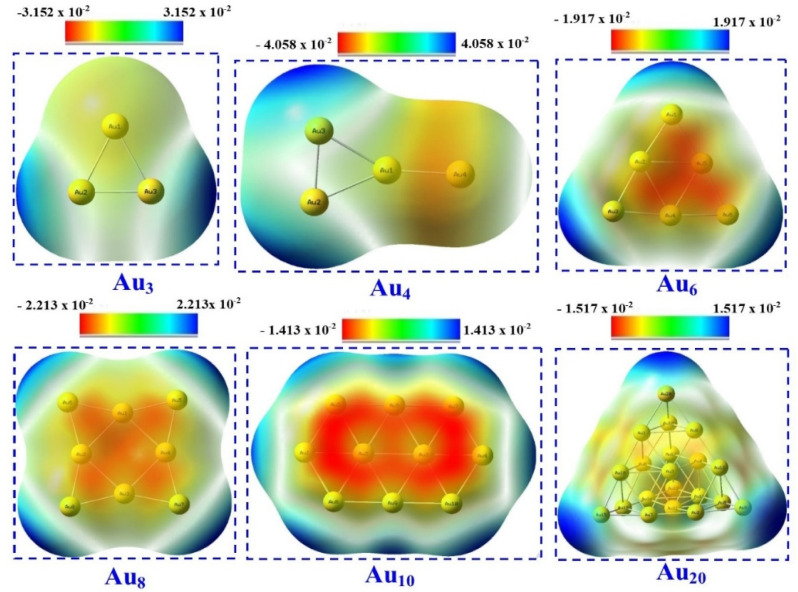
MEP plots of Au_*n*_NPs (*n* = 3, 4, 6, 8, 10, and 20) in gas phase.

Maps of molecular electrostatic potential (MEP) show the surface-based electric charge distribution on a molecule. For Au_*n*_ (*n* = 3, 4, 6, 8, 10, and 20), MEP helps one see areas displaying positive and negative charges. Au_3_NPs lack negative potential; instead, a positive area is found at both extreme sides of the base of the equilateral triangle form (Au_2_ and Au_3_), where the molecular electrostatic potential in these sites was expected to be around −3.152 × 10^−2^ (a.u). This discovery is validated by the values of MEP quantum molecular descriptors. Whereas the negative region is situated between the apex of Au_1_ in the equilateral triangle and Au_4_, where the molecule electrostatic potential in these areas was computed to be approximately −4.058 × 10^−2^ (a.u), the positive regions in Au_4_ nanoparticles are found at the extreme ends of the triangle's base (Au_2_ and Au_3_). Whereas the negative region is located within the triangle, where the molecular electrostatic potential in these places was computed to be about −1.917 × 10^−2^ (a.u), the positive regions in Au_6_ nanoparticles are found at the vertices of the triangle (Au_3_, Au_6_, and Au_1_). The positive regions of Au_8_ nanoparticles are found at the extreme corners of the square (Au_8_, Au_6_, Au_5_, and Au_7_), whilst the negative zone is situated inside the square where the electrostatic potential of the molecule in these areas was computed to be around −2.213 × 10^−2^ (a.u). Whereas a negative zone exists inside the hexagonal structure, where the molecule electrostatic potential in these places was computed to be around −1.414 × 10^−2^ (a.u), positive regions in Au_10_NPs are found at the outermost edges of each exterior AuNP (Au_1_, Au_5_, Au_6_, Au_7_, Au_4_, Au_8_, Au_9_, and Au_10_). The positive portions of Au_20_NPs are found at the extreme borders of the tetrahedron. The negative region is confined inside the structure in negligible concentration, where the electrostatic potential of the molecule in these sites was computed to be around −1.517 × 10^−2^ (a.u). Red denotes areas of high electron density while blue denotes areas with reduced electron density; this helps one to visually identify charge distribution throughout the molecule surface as seen in Fig. 6.

#### 3.4.3. Thermodynamic parameters of Au_*n*_NPs (*n* = 3, 4, 6, 8, 10 and 20)

Table 2 presents thermodynamic characteristics, which indicate the thermal stability of a molecule, thereby helps in predicting spontaneous reactions. A reaction proceeds on its own when the Gibbs free energy (Δ*G*) is negative. Regarding Au_*n*_NPs (*n* = 3, 4, 6, 8, 10, and 20), clusters show negative values; nonetheless, Au_20_NPs have a larger value than the other selected AuNPs. As the enthalpy (Δ*H*) lowers in an exothermic process, heat energy is discharged into the surroundings. With *n* = 3, 4, 6, 8, 10, and 20, Au_20_NPs have the highest value among Au_*n*_NPs. There are notable differences in the properties; negative values point to exothermic, spontaneous, and orderly interactions. The negative values of these interactions suggest that they happen independently and cause heat emission. Estimating enthalpy of formation depends on the zero-point vibrational energy (ZPVE) for Au_*n*_NPs; so, for clusters in the gas phase, respectively, the values 0.346, 0.737, 1.451, 2.000, 2.533, and 5.620 kcal mol^−1^ have been found. Furthermore calculated are the Gibbs free energy values of the most stable nanocomplexes to determine if the clusters show exothermic or endothermic properties. Indicating that the interaction is exothermic due to the negative values, the estimated Gibbs free energy values for Au_*n*_NPs clusters (*n* = 3, 4, 6, 8, 10, and 20) in the gas phase are −406.4, −541.9, −812.9, −1083.9, −1354.9, and −2710.1 kcal mol^−1^, respectively.

**Table 2:** Thermodynamic quantities properties of Au_*n*_NPs (*n* = 3, 4, 6, 8, 10 and 20) in gaseous phase computed at B3LYP/LANL2DZ

Property	Clusters					
	Au_3_	Au_4_	Au_6_	Au_8_	Au_10_	Au_20_
Energy Δ*E* (Hartree)	−406.4	−541.9	−812.9	−1083.9	−1354.9	−2710.1
Enthalpy Δ*H* (Hartree)	−406.4	−541.9	−812.9	−1083.9	−1354.9	−2710.1
Gibbs free energy Δ*G* (Hartree)	−406.4	−541.9	−812.9	−1083.9	−1354.9	−2710.1
Energy Δ*E* (kcal mol^−1^)	3.00	5.4	9.02	12.6	16.2	34.2
Specific heat capacity cv (cal mol^−1^-kelvin)	9.82	17.6	29.4	41.1	53.0	111.9
Entropy S (cal mol^−1^-kelvin)	83.39	106.7	133.3	168.7	195.9	328.3
Zero-point vibrational energy (ZPVE) (kcal mol^−1^)	0.346	0.737	1.451	2.000	2.533	5.620

#### 3.4.4. Analysis of the ELF and LOL

The topological analysis surface is the main presentation of the ELF and LOL analysis, which also shows a high likelihood of finding an electron pair on the molecular surface. To do this, the Multiwfn software^37^ package is used. Since LOL and ELF both rely on the kinetic energy density, their chemical mappings can be mathematically compared. The locations of atomic shells, lone pairs, and core binding electrons in atomic and molecular orbitals may be clearly indicated by the ELF and LOL functions. The current molecules' chemical bond is represented graphically by this dimensionless number, which goes from 0 to 0.8 in LOL and 0 to 1 in ELF.

**Fig. 7:**
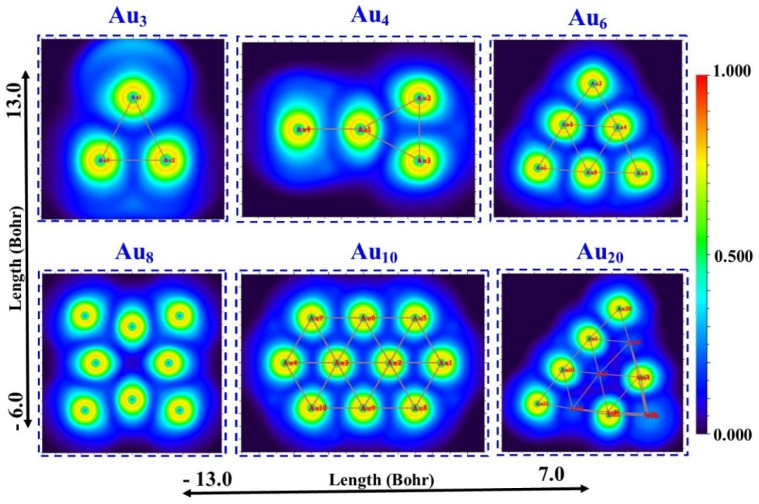
ELF colour-filled plots of Au_*n*_NPs (*n* = 3, 4, 6, 8, 10, and 20) in the gas phase.

**Fig. 8:**
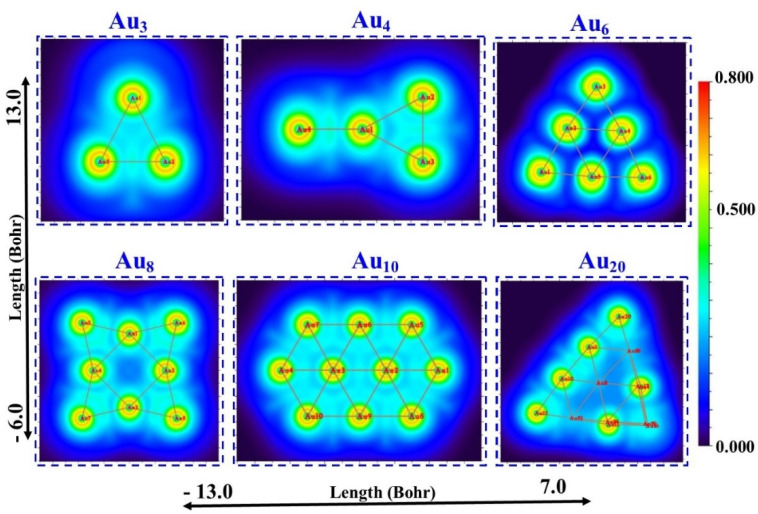
LOL colour-filled plots of Au_*n*_NPs (*n* = 3, 4, 6, 8, 10, and 20) in the gas phase.

The electron distribution of Au_*n*_NPs(*n* = 3, 4, 6, 8, 10 and 20) clusters is displayed in Fig. 7, which is particularly useful for locating areas of significant electron localization.^48^ The blue areas surrounding Au in all clusters in ELF (Fig. 7) represent the delocalized electron cloud.Fig. 8 The range of the ELF and LOL values (Bohr) is −13.0 to 7.0.

Although our work focuses on AuNPs, the reduced energy gaps and enhanced charge-transfer features obtained from DFT show reactivity trends similar to those reported for AgNPs, thereby explaining the efficient ROS-mediated dye degradation and antibacterial activity observed in our gold nanomaterials.

### 3.5. Machine learning analysis

The use of machine learning (ML) for bandgap energy prediction offers significant advantages in materials science and semiconductor research. Traditionally, determining bandgap energy requires extensive experimental procedures, which are time-consuming and resource-intensive. By leveraging ML models like XGBoost, LightGBM, and Neural Networks, researchers can achieve fast, accurate, and scalable predictions without the need for costly experiments. Machine leaning model performance and respective table provided in Supplementary file.

#### 3.5.1. Visual analysis of bandgap

**Fig. 9:**
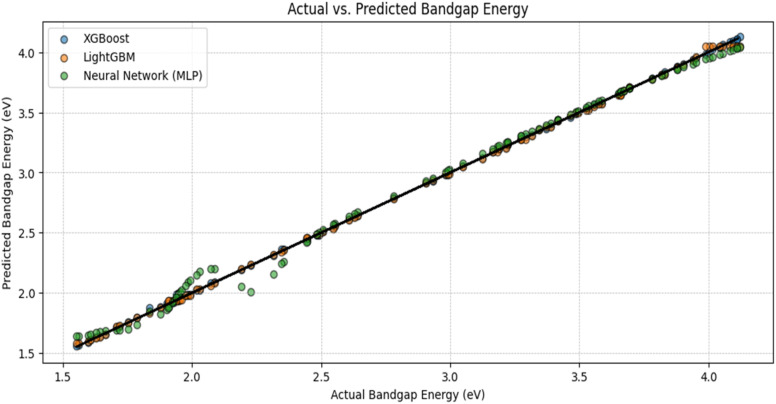
Experimental *vs.* ML-Predicted Bandgap (Scatter Graph).

**Fig. 10:**
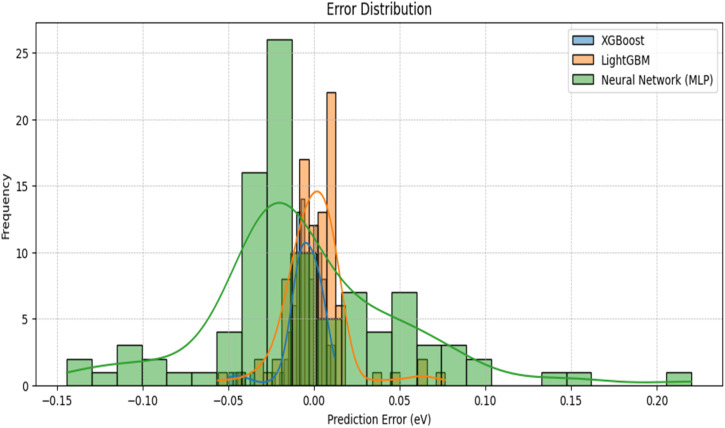
Error distribution graph.

A scatter plot (Fig. 9) was generated to compare the actual *vs.* predicted bandgap energy values for all models. The predictions from XGBoost and LightGBM closely followed the perfect prediction line, confirming their reliability. The error distribution analysis (Fig. 10) showed that XGBoost and LightGBM had a narrow error spread, indicating better predictive accuracy. The Neural Network model had a slightly wider error spread, suggesting potential improvements with more data or tuning.

#### 3.5.2. SHAP analysis

**Fig. 11:**
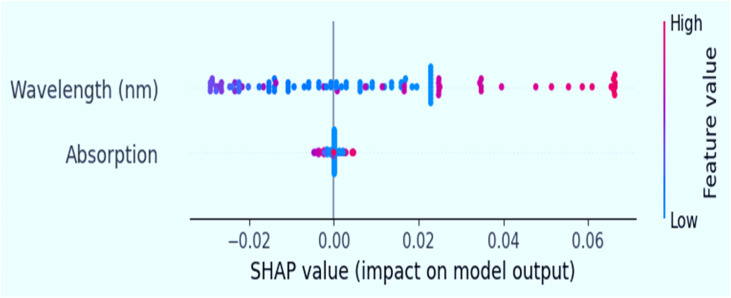
SHapley Additive exPlanations (SHAP) analysis on the best-performing model XGBoost.

To gain deeper insights into model decision-making, we performed SHAP (SHapley Additive exPlanations) analysis on the best-performing model (XGBoost) were shown in Fig. 11. To interpret the machine learning model's decision-making process, SHapley Additive exPlanations (SHAP) analysis was conducted. The results highlight the relative contributions of wavelength (nm) and absorption features to the predicted bandgap energy. SHAP dependent plot and their summery discussed in supplementary file.

## 4. Conclusions

In summary, AuNPs was successfully synthesized using the leaf extract of *Acacia nilotica*, which act as a stabilizing as well as reducing agent to control the growth of particles. As synthesized AuNPs was characterized by using spectroscopic and microscopic techniques. The existence of characteristic peaks in spectrometry techniques corroborates the formation of AuNPs. The formation of a smaller-sized and spherical-shaped AuNPs with a high surface area acted as an effective photocatalyst promoting degradation (92.5%) within a very short reactive time (10 min) of MO under visible light irradiation. The investigated AuNP catalyst was suitable for practical application in MO treatment as it showed higher activity after the 4th cycle. This investigation examines the use of Au_*n*_NPs (*n* = 3, 4, 6, 8,10 and 20) in photocatalytic process by DFT. This investigation focuses on various key parameters that includes, Fermi level, HOMO–LUMO energy gap, work function, topological (ELF and LOL) and thermal stability. DFT computations have been utilized to investigate the optimized structures of Au_*n*_NPs (*n* = 3, 4, 6, 8, 10 and 20). The electrical conductivity of Au_*n*_NPs has been found to vary as a result of a significant decrease in their band gap. Moreover, Gibbs (free) energies in the gas phase have been found negative showing an exothermic process. The chemical implication of Au_*n*_NPs was explained using ELF and LOL analysis gives information about interactions. We found a high-correlation coefficient of 0.9878% between DFT provided bandgap and predicted bandgap results with high prediction accuracy as MSE of 0.00035. Additionally, Shapley Additive exPlanations (SHAP) are also discussed.

## Author contributions

M. Yasmin Begum, Mukta Sharma, M. V. Arularasu, P. Vinitha, V. Vetrivelan: synthesize and characterize the samples, writing, graphical, and software work, construction plan, writing– original draft preparation, visualization, format checking. Geetha Kandasamy, Manikandan Ayyar, Abinet Gosaye Ayanie, Lalitha Gnanasekaran: rewriting, reviewing the manuscript and format checking, revision of the paper and discussion. Ankush Mehta, Rupesh Gupta: reviewing the manuscript and format checking, revision of the paper and discussion.

## Conflicts of interest

We declare that we have no known competing financial interests or personal relationships that could have appeared to influence the work reported in this paper.

## Data Availability

The data supporting the findings of this study are available from the corresponding author upon reasonable request.
